# Healthcare providers consistently overestimate the diagnostic probability of ventilator-associated pneumonia

**DOI:** 10.1017/ice.2023.62

**Published:** 2023-12

**Authors:** Nathaniel S. Soper, Owen R. Albin

**Affiliations:** Department of Internal Medicine, University of Michigan, Ann Arbor, Michigan

## Abstract

**Objective:**

To assess the accuracy of provider estimates of ventilator-associated pneumonia (VAP) diagnostic probability in various clinical scenarios.

**Design:**

We conducted a clinical vignette-based survey of intensive care unit (ICU) physicians to evaluate provider estimates of VAP diagnostic probability before and after isolated cardinal VAP clinical changes and VAP diagnostic test results. Responses were used to calculate imputed diagnostic likelihood ratios (LRs), which were compared to evidence-based LRs.

**Setting:**

Michigan Medicine University Hospital, a tertiary-care center.

**Participants:**

This study included 133 ICU clinical faculty and house staff.

**Results:**

Provider estimates of VAP diagnostic probability were consistently higher than evidence-based diagnostic probabilities. Similarly, imputed LRs from provider-estimated diagnostic probabilities were consistently higher than evidence-based LRs. These differences were most notable for positive bronchoalveolar lavage culture (provider-estimated LR 5.7 vs evidence-based LR 1.4; *P* < .01), chest radiograph with air bronchogram (provider-estimated LR 6.0 vs evidence-based LR 3.6; *P* < .01), and isolated purulent endotracheal secretions (provider-estimated LR 1.6 vs evidence-based LR 0.8; *P* < .01). Attending physicians and infectious disease physicians were more accurate in their LR estimates than trainees (*P* = .04) and non-ID physicians (*P* = .03).

**Conclusions:**

Physicians routinely overestimated the diagnostic probability of VAP as well as the positive LRs of isolated cardinal VAP clinical changes and VAP diagnostic test results. Diagnostic stewardship initiatives, including educational outreach and clinical decision support systems, may be useful adjuncts in minimizing VAP overdiagnosis and ICU antibiotic overuse.

Treatment for respiratory infections including ventilator-associated pneumonia (VAP) is responsible for the majority of antibiotic use in intensive care units (ICUs).^1^ However, accurately diagnosing VAP is challenging and misdiagnosis occurs in up to 60% of cases.^2^ VAP overdiagnosis generates excessive antibiotic use, catalyzing acquisition and spread of drug-resistant organisms and generating adverse drug events including *Clostridioides difficile* infection and nephrotoxicity.^3,4^

Because VAP diagnosis lacks a gold-standard confirmatory test, providers frequently base treatment decisions on bedside estimates of diagnostic likelihood, summated from combinations of nonspecific clinical, radiographic, and microbiologic findings. However, clinical providers often struggle with probabilistic reasoning and are susceptible to a multitude of cognitive biases.^5^ This results in overestimation of disease probability and a misunderstanding of the implications of positive test results for commonly encountered conditions.^5^

Given the prevalence of VAP overdiagnosis and its effect on excess ICU antibiotic use, understanding how practitioners approach diagnostic reasoning in suspected VAP is of critical importance. We assessed the accuracy of ICU practitioner estimates of VAP diagnostic probability before and after isolated cardinal VAP clinical changes and VAP diagnostic test results relative to evidence-based estimates of VAP diagnostic probability.

## Methods

We performed a single-center survey of ICU providers at Michigan Medicine University Hospital, a tertiary-care center with 7 ICUs and 108 ICU beds. The University of Michigan Institutional Review Board approved this study for waiver of informed consent.

### Survey

We developed a web-based survey to evaluate ICU practitioner estimates of diagnostic probability of VAP in response to commonly encountered cardinal VAP clinical changes and VAP diagnostic test results in critically ill patients. A draft survey was developed by the primary investigators and iteratively reviewed by experts in infectious diseases, critical care medicine, and qualitative research methodology.

Survey respondents were sequentially presented with 5 clinical vignettes describing a mechanically ventilated patient with a new isolated cardinal VAP clinical change (ie, fever, leukocytosis, increased oxygenation requirements, purulent endotracheal secretions, or increased nonpurulent endotracheal secretions), followed by a subsequent VAP diagnostic test result (ie, chest radiograph with or without an opacity, positive or negative respiratory culture [endotracheal or bronchoalveolar lavage], or positive respiratory sample gram stain). Respondents were asked to estimate the probability of VAP on a scall of 0%–100% after each isolated cardinal VAP clinical change and following each VAP diagnostic test result. Full details of survey design and a sample clinical vignette are provided in the Supplementary Materials (online).

### Recruiting and enrollment

Survey respondents were recruited among clinical faculty and house staff at the University of Michigan. The survey was distributed via institutional email lists to 509 physicians, fellows, and residents in the fields of internal medicine, cardiology, pulmonology and critical care, infectious disease, general surgery, and anesthesiology. Participation was incentivized using gift cards. We sent 2 weekly email reminders to complete the survey.

### Statistical analysis

Provider estimates of VAP prevalence for a standard patient on mechanical ventilation for >48 hours were used as surrogate estimates of VAP baseline probability. For each vignette, we defined provider-estimated VAP pre-test probability as a given respondent’s estimated probability of disease following development of a new isolated cardinal VAP clinical change but prior to a VAP diagnostic test result. Provider-estimated VAP posttest probability was defined as a respondent’s subsequent estimated probability of disease following a VAP diagnostic test result.

Provider-estimated baseline, pretest, and posttest VAP probabilities were compared to evidence-based VAP diagnostic probabilities. We employed an evidence-based baseline VAP probability of 16% (8.0%–24.0%), derived from observational studies and randomized controlled trials (Supplementary Materials online). We then applied evidence-based likelihood ratios (LRs) obtained from autopsy-based systematic review and meta-analyses to each vignette prompt to calculate evidence-based pretest probabilities for each new isolated cardinal VAP clinical change and evidence-based posttest probabilities for each VAP diagnostic test result (Supplementary Tables 1 and 2 online).^6,7^ Provider-specific imputed LRs were calculated for each vignette prompt and compared to evidence-based LRs by dividing posttest odds by pretest odds, where odds were calculated as probability divided by 1 minus probability. Full details on calculations of provider-estimated and evidence-based pre- and posttest probabilities are provided in the Appendix (online).

All statistical analyses were performed using SAS version 9.4 software (SAS Institute, Cary, NC). Descriptive statistics were calculated for provider-estimated baseline, pretest, and posttest probabilities as well as imputed LRs. Provider estimates of VAP diagnostic probability for each vignette prompt were visualized using density plots. Wilcoxon signed-rank tests were used to compare distributions of provider-estimated and evidence-based diagnostic probabilities. Provider-estimated and evidence-based LRs were log transformed and compared using Wilcoxon signed-rank tests. Prespecified respondent subgroup analyses were performed, stratified by training level (attending physician vs trainee), experience level (>10 years vs <10 years ICU experience) and specialty (internal medicine vs non–internal medicine, infectious disease vs other). For subgroup comparisons, the overall mean difference between provider-estimated and evidence-based LRs across all case vignettes were compared using *t* tests.

## Results

### Participant demographics

We collected 133 survey responses, a survey response rate of 26%. Overall, 30% of respondents were attending physicians and 70% were residents or fellows (see Table 1). Also, 71% of respondents were in the field of internal medicine or related subspecialties, including 19% of respondents from the field of infectious disease. Furthermore, 49% of respondents reported ≤2 years of ICU practice experience whereas 18% of respondents reported >10 years of experience. Finally, 66% of survey respondents reported being moderately to extremely confident in their ability to accurately diagnose VAP.

**Table 1.** Demographic Data of Survey Respondents

Demographics		Respondents, No. (%)
Provider specialty	General internal medicine	35 (33.9)
	Infectious diseases	20 (19.4)
	Pulmonary & critical care medicine	17 (16.5)
	Cardiology	4 (3.9)
	Anesthesiology	10 (9.7)
	General surgery	11 (10.7)
	Neurology	6 (5.8)
Training level	Resident	59 (56.2)
	Fellow	14 (13.3)
	Attending	32 (30.5)
Experience^a^	0–2 y	50 (48.1)
	3–5 y	24 (23.1)
	6–10 y	11 (10.6)
	>10 y	19 (18.3)
Confidence in diagnosing VAP	Not at all confident	8 (10.7)
	Not very confident	26 (34.7)
	Moderately confident	20 (26.7)
	Very confident	21 (28.0)
	Extremely confident	0 (0)

a Years of experience refers to number of years working in a clinical ICU setting.

### Estimates of VAP diagnostic probability

**Figure 1.**
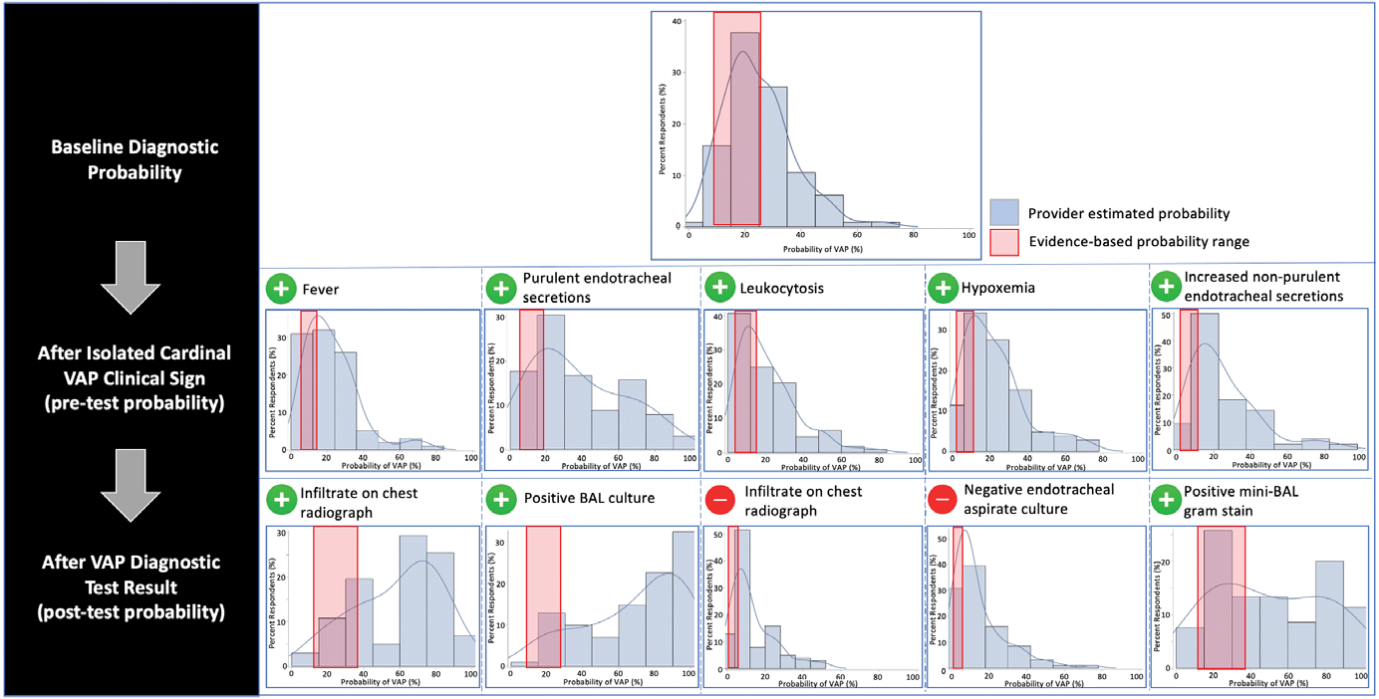
Density plots of provider-estimated vs evidence-based diagnostic probability of ventilator-associated pneumonia. Density plots depicting provider-estimated diagnostic probability of VAP at baseline, after development of an isolated cardinal VAP clinical sign, and following a subsequent VAP diagnostic test result for each vignette prompt. Area highlighted in red represents the evidence-based range of diagnostic probability. Note. VAP, ventilator-associated pneumonia. BAL, bronchoalveolar lavage. Mini-BAL refers to nonbronchoalveolar lavage.

Provider estimates of VAP probability were consistently higher than evidence-based probabilities both at baseline and following clinical vignette prompts (Fig. 1). Survey respondents overestimated the baseline probability of VAP with a median estimated probability of 20% (interquartile range [IQR], 15–30) relative to evidence-based baseline probability of 16% (*P* < .01). Provider-estimated VAP probabilities were similarly overestimated following presented isolated cardinal VAP clinical changes and VAP diagnostic test results. For example, the median provider-estimated pretest probability of VAP following patient development of isolated purulent endotracheal secretions was 34% (IQR, 19–62) relative to an evidence-based pre-test probability of 12.6% (*P* < .01). In the same clinical vignette, following receipt of a positive bronchoalveolar lavage (BAL) culture, the median provider-estimated post-test VAP probability rose to 80% (IQR, 50–92) relative to an evidence-based post-test probability of 16.5% (*P* < .01).

### Imputed likelihood ratios

**Figure 2.**
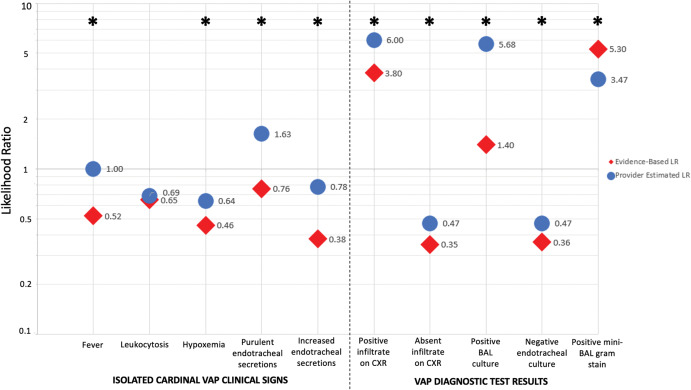
Provider-estimated versus evidence-based diagnostic likelihood ratios for clinical signs and test results for ventilator-associated pneumonia. Scatterplot showing the differences in median provider-estimated diagnostic likelihood ratios relative to evidence-based likelihood ratios. Note. CXR, chest radiograph. BAL, bronchoalveolar lavage. “Positive infiltrate on CXR” refers to a chest radiograph with an opacity with an air bronchogram. *Denotes statistically significant differences (*P* < .05).

Imputed likelihood ratios calculated from respondent pretest and posttest probabilities consistently overestimated the impact of isolated cardinal VAP clinical changes and VAP diagnostic test results on VAP diagnostic likelihood (Fig. 2). Overall, the mean difference between provider-estimated LRs and evidence-based LRs was largest with positive BAL culture (median provider-estimated LR 5.68 vs evidence-based LR 1.40; *P* < .01) and chest radiograph with air bronchograms (median provider-estimated LRs 6.00 vs evidence-based LR 3.80; *P* < .01). Of the 10 vignette prompts included in the survey, providers overestimated the LR in 9 instances. The only prompt for which providers underestimated the evidence-based LR was positive BAL gram stain (median provider-estimated LR 3.47 vs evidence based LR 5.30; *P* = .03).

Among the presented isolated cardinal VAP clinical changes, the largest discrepancies between provider-estimated and evidence-based LRs were noted for fever (median provider-estimated LR 1.00 vs evidence-based LR 0.52; *P* < .01) and purulent endotracheal secretions (median provider-estimated LR 1.63 vs evidence-based LR 0.76; *P* < .01). Notably, provider-estimated imputed LRs for leukocytosis were relatively consistent with evidence-based values (median provider-estimated LR 0.69 vs evidence-based LR 0.65; *P* = .52).

### Subgroup analyses

Trainees significantly overestimated baseline VAP diagnostic probability (median provider-estimated baseline probability 25% vs evidence-based baseline probability 16%; *P* < .01), whereas attending physicians did not (provider-estimated 20% vs evidence-based 16%; *P* = .06). Comparing mean provider-estimated LRs versus evidence-based LRs, attending physicians were also significantly more likely to accurately estimate LRs for isolated cardinal VAP clinical changes and VAP diagnostic test results (*P* < .01). Similarly, infectious disease physicians were more accurate in LR estimations relative to non–infectious disease physicians (*P* = .03). We detected a trend toward improved accuracy of estimated LRs among providers with >10 years ICU experience compared to those with <10 years of experience (*P* = .07). We did not detect significant differences when comparing internal medicine providers to other providers or when comparing those with high self-described confidence in diagnosing VAP to those with low confidence.

## Discussion

In this survey study of 133 ICU clinical providers at various levels of training, physicians consistently overestimated the baseline probability of VAP, pretest probabilities of VAP following isolated clinical changes, and posttest probabilities of VAP following performance of common VAP diagnostic tests. Provider-estimated imputed likelihood ratios for isolated cardinal clinical changes and diagnostic test results for VAP were also consistently higher than evidence-based reference values. Providers most significantly overestimated the importance of positive respiratory cultures, chest radiographs with air bronchograms, and the presence of purulent endotracheal secretions in determining diagnostic probability of VAP.

Our findings are concordant with those of prior studies showing that practitioners consistently overestimate the likelihood of disease both at baseline and in response to clinical changes and test results.^8^ For VAP, overestimating diagnostic probability after a patient develops an isolated cardinal clinical change (eg, a fever or a change in endotracheal secretions) may invite unnecessary diagnostic testing (eg, respiratory culturing or radiography), the diagnostic utility of which is also overestimated. Cascading overestimations of diagnostic likelihood may lead to unnecessary antibiotic use for an unlikely diagnosis of VAP.

Most efforts to reduce antibiotic overuse in VAP have historically focused on therapeutic processes, such as antibiotic de-escalation and standardizing treatment durations for confirmed VAP cases.^9^ Although interventions at this stage have been modestly effective, they face challenges including antibiotic inertia (eg, difficulty of discontinuing antibiotics after they have been started).^9,10^ Comparatively fewer studies have examined diagnostic stewardship strategies for VAP antibiotic overuse—interventions at the level of diagnostic test ordering, collection, and reporting that can safely reduce ICU antibiotic overuse.

Misinterpretation of the diagnostic importance of isolated cardinal VAP clinical changes and VAP diagnostic test results demonstrated by this study suggest that diagnostic stewardship interventions targeting the ordering phase of VAP diagnostic testing may be beneficial in reducing unnecessary antibiotic use. As a comparison, antibiotic overuse in asymptomatic bacteriuria has been driven by misconceptions regarding the specificity of clinical changes such as altered mental status in cognitively or functionally impaired individuals and test results including bacteriuria.^11^ Educational interventions addressing this misunderstanding have been effective in reducing unnecessary testing and antibiotic use in this setting, especially when paired with other antimicrobial stewardship interventions such as formal diagnostic guidelines.^12,13^ Similar educational efforts may be effective as part of diagnostic stewardship efforts for VAP by emphasizing the poor specificity of isolated clinical changes, such as fever or purulent endotracheal secretions and the prevalence of endotracheal bacterial colonization. The improved performance of attending physicians relative to trainees is suggestive of the benefits of additional education and experience in accurately diagnosing VAP. Given discrepancies noted between attending physician and trainee overestimation of diagnostic likelihood in this study, trainees may be a high-yield target for educational interventions because they often serve as frontline providers ordering point-of-care diagnostic testing.

Prior studies demonstrate that providers often struggle with the clinical application of Bayesian reasoning.^14^ Given the complexity of VAP diagnosis, the use of clinical decision support systems (CDSSs) to guide diagnostic and treatment decisions may be beneficial to decrease unnecessary diagnostic testing. CDSSs are defined as computer applications designed to assist clinical decision making by analyzing data within electronic medical records and providing prompts and reminders to optimize evidence-based decisions at the point of care. CDSSs have already been shown to be widely beneficial in antimicrobial stewardship efforts and reducing unnecessary testing but remain unexplored in VAP.^15,16^ CDSSs providing VAP diagnostic probabilities based on available clinical data may help reduce unnecessary culturing and subsequent overtreatment related to misinterpreted positive respiratory cultures in patients with low pretest probability for VAP.^17^

This study had several limitations. The LRs used for evidence-based isolated cardinal VAP clinical changes and VAP diagnostic test results were obtained from autopsy-based studies, which are subject to spectrum bias; thus, their accuracy is uncertain. Furthermore, as a single-center study with a limited response rate, our findings may not be generalizable, especially given the extent to which institutional culture influences diagnostic testing and antibiotic use. Lastly, the differences observed in this study between provider-estimated and evidence-based VAP diagnostic probabilities may not be clinically meaningful because decisions underlying real-world antimicrobial use in critically ill patients are complex and are only partially based on precise appraisals of diagnostic likelihood.

In summary, these results have demonstrated that ICU physicians routinely overestimate the diagnostic probability of VAP and the positive likelihood ratios of commonly encountered isolated VAP cardinal signs and symptoms, as well as radiographic and microbiologic test results. Thus, diagnostic stewardship initiatives, including educational outreach and clinical decision support systems, may be useful adjunctive tools in minimizing ICU antibiotic overuse.

## Supplementary material

For supplementary material accompanying this paper visit https://doi.org/10.1017/ice.2023.62. click here to view supplementary material
