# Supplementary material for: Healthcare providers consistently overestimate the diagnostic probability of ventilator-associated pneumonia
Source: Infect Control Hosp Epidemiol. 2023 Jun 23;44(12):1927–31. doi: 10.1017/ice.2023.62 (PMC10755149; doi:10.1017/ice.2023.62)
Supplement: Supplementary file 1 [file S0899823X23000624sup001.docx]

**APPENDIX:**

Survey Description

The survey captured basic demographic information including training background, years of ICU experience, and self-reported confidence in VAP diagnosis (the latter evaluated on a custom ordinal scale). Survey respondents were asked to estimate the frequency of VAP in patients requiring mechanical ventilation for >48 hours, which was used as a surrogate estimate of baseline VAP diagnostic probability. Respondents were then sequentially presented with five clinical vignettes. Each clinical vignette provided a patient age, indication for mechanical ventilation, and duration of mechanical ventilation. The vignette then presented a new isolated cardinal VAP clinical change (fever, leukocytosis, increased oxygenation requirements, purulent endotracheal secretions, or increased non-purulent endotracheal secretions). Respondents were asked to estimate the diagnostic probability of VAP on a scale of 0-100% in response to these clinical changes. For each isolated cardinal VAP clinical change, it was emphasized that the described clinical change (e.g. fever) occurred in the absence of the other potential clinical changes (i.e. leukocytosis, increased oxygenation requirements, or change in endotracheal secretions) to isolate the perceived diagnostic utility of each individual clinical change. After this, respondents were presented with a VAP diagnostic test result (chest radiograph with or without an opacity, positive or negative respiratory culture [endotracheal or bronchoalveolar lavage], or positive respiratory sample gram stain). Respondents were again asked to estimate the probability of VAP following VAP diagnostic test results. Supplemental Figure 1 provides an example of one of the cases included on the survey. Provider estimates of VAP diagnostic likelihood were captured on a scale of 0-100%. Responses of 0% or 100% were modified to 0.1% or 99.9% to facilitate statistical analysis.

Determination of Evidence-Based Diagnostic Probabilities

The baseline diagnostic probability range for ventilator-associated pneumonia (VAP) was derived from review of studies in which incidence of VAP was reported. Studies were identified through a PubMed search using the terms “ventilator-associated pneumonia” and “epidemiology”, “incidence”, or “rates”. The identified studies included several observational cohort and cross-sectional studies, as well as randomized controlled trials from which the control group was referenced^1–9^. Determination of a precise range was limited by the variety of case definitions used in different studies. Studies using combined clinical, microbiological, and radiographic diagnostic criteria were prioritized. Studies involving high-risk patient populations (those with Acute Respiratory Distress Syndrome, receiving Extracorporeal Membrane Oxygenation, and intracranial hemorrhage/traumatic brain injury) were excluded. Overall, it was determined that studies suggest a VAP prevalence of 8.0 - 24.0% among average risk mechanically ventilated patients. For statistical comparisons, a midpoint prevalence of 16.0% was used.

Evidence-based likelihood ratios were obtained from autopsy-based systematic reviews and meta-analyses (see Appendix Table 1 & 2)^10,11^. Evidence-based pre-test probabilities for each isolated cardinal VAP clinical change were calculated using the evidence-based positive likelihood ratio (LR) for the present clinical change and the negative likelihood ratios of the absent other three clinical changes. For example, for the first clinical vignette in which the vignette prompt describes a new isolated fever, the positive likelihood ratio for fever was applied to the evidence-based baseline diagnostic probability as well as the negative likelihood ratios for leukocytosis, hypoxia, and purulent endotracheal secretions. For this calculation, baseline diagnostic probability was converted to baseline odds, calculated as probability divided by 1 minus probability. Then, the pertinent likelihood ratios were applied to the evidence-based baseline odds sequentially. The resultant combined LRs for each vignette prompt, a combination of the relevant positive and negative LRs, are shown in Supplemental Table 1. For the clinical change of increased non-purulent endotracheal secretions, the combined LR reflects the evidence based negative LRs for fever, leukocytosis, hypoxemia, and purulent endotracheal secretions were used as there is no data establishing positive or negative LRs for this clinical change. For each VAP diagnostic test result provided in the clinical vignettes, evidence-based post-test probabilities were calculated by applying the relevant positive or negative likelihood ratio to the pre-test probability calculated for that vignette.

Calculation of Imputed Provider-Specific Likelihood Ratios

To understand providers’ perceived change in probability of disease after isolated cardinal VAP clinical changes and VAP diagnostic test results, and to account for the fact that inflated pre-test estimates of VAP diagnostic probability can affect post-test estimates, we calculated provider-specific imputed LRs for each vignette prompt. The imputed LR was calculated by dividing post-test odds by pre-test odds, where odds were calculated as probability divided by 1 minus probability. Provider estimated LRs for each prompt were compared to evidence-based LRs.

**Supplemental Table 1.** Evidence-Based Positive and Negative Likelihood Ratios and Imputed Likelihood Ratios for Isolated Clinical Changes Used for Statistical Analysis

| **Isolated Cardinal VAP Clinical Change** | **Positive Likelihood Ratio** | **Negative Likelihood Ratio** | **Combined Likelihood Ratio for Isolated Change** |
| --- | --- | --- | --- |
| Fever | 1.2 | 0.86 | 0.52 |
| Leukocytosis | 1.3 | 0.74 | 0.65 |
| Hypoxia | 1.1 | 0.9 | 0.46 |
| Purulent Endotracheal Secretions | 1.3 | 0.63 | 0.76 |
| Increased Non-purulent Endotracheal Secretions | N/A | N/A | 0.38 |

**Supplemental Table 2.** Evidence-Based Positive and Negative Likelihood Ratios for VAP Diagnostic Test Results

| **VAP Diagnostic Test Result** | **Positive Likelihood Ratio** | **Negative Likelihood Ratio** |
| --- | --- | --- |
| Chest radiograph with a single air bronchogram | **3.80** | 0.87 |
| Chest radiograph with any infiltrate | 1.70 | **0.35** |
| Endotracheal culture with >105 CFUs | 2.36 | **0.36** |
| Bronchoalveolar lavage culture with >104 CFUs | **1.40** | 0.78 |
| Mini-BAL gram stain | **5.30** | 0.50 |

CFUs = colony forming units. BAL = bronchoalveolar lavage. The LR values used in the study are in bold.

**Supplemental Figure 1**. Example of Clinical Vignette from Digital Survey


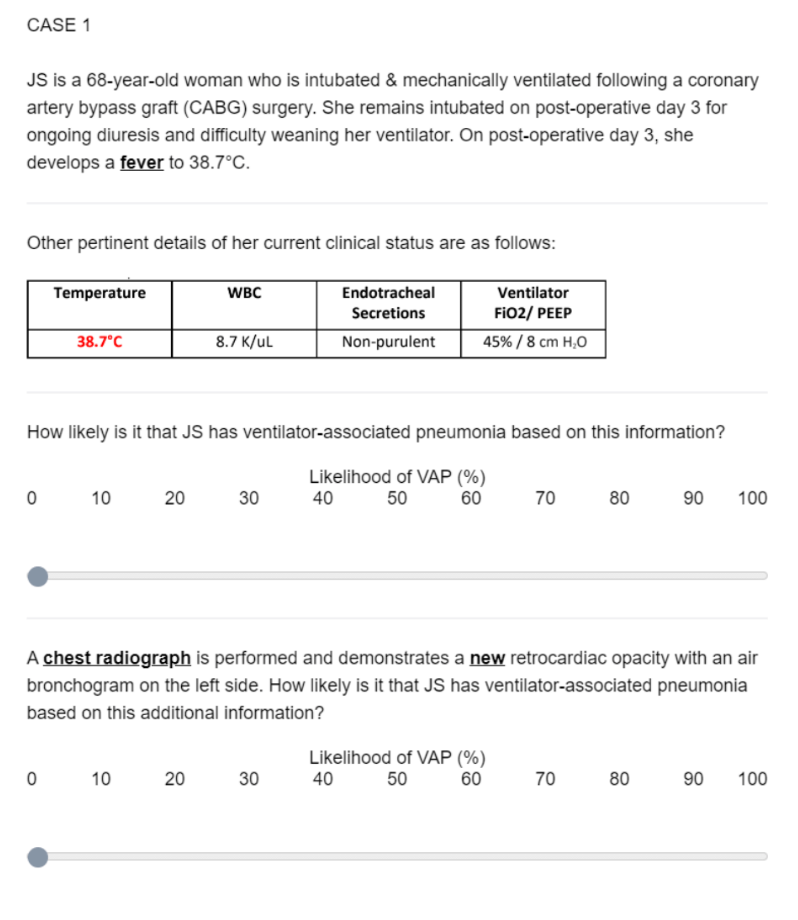


Supplemental Figure 1. Excerpt from digital survey demonstrating clinical vignette case 1. Respondents used a sliding bar to select diagnostic probability following an isolated cardinal VAP clinical change and a subsequent VAP diagnostic test result.

**REFERENCES:**

1. Cook, D. J. *et al.* Incidence of and risk factors for ventilator-associated pneumonia in critically ill patients. *Ann Intern Med* **129**, 433–440 (1998).

2. Martin-Loeches, I. *et al.* Incidence and prognosis of ventilator-associated tracheobronchitis (TAVeM): a multicentre, prospective, observational study. *Lancet Respir Med* **3**, 859–868 (2015).

3. Koulenti, D., Tsigou, E. & Rello, J. Nosocomial pneumonia in 27 ICUs in Europe: perspectives from the EU-VAP/CAP study. *European Journal of Clinical Microbiology and Infectious Diseases* **36**, 1999–2006 (2017).

4. Metersky, M. L. *et al.* Trend in Ventilator-Associated Pneumonia Rates Between 2005 and 2013. *JAMA* **316**, 2427–2429 (2016).

5. Wałaszek, M., Rózańska, A., Wałaszek, M. Z. & Wójkowska-Mach, J. Epidemiology of Ventilator-Associated Pneumonia, microbiological diagnostics and the length of antimicrobial treatment in the Polish Intensive Care Units in the years 2013-2015. *BMC Infect Dis* **18**, (2018).

6. Bhadade, R., Harde, M., DeSouza, R., More, A. & Bharmal, R. Emerging Trends of Nosocomial Pneumonia in Intensive Care Unit of a Tertiary Care Public Teaching Hospital in Western India. *Ann Afr Med* **16**, 107 (2017).

7. Takahama, A. *et al.* Analysis of oral risk factors for ventilator-associated pneumonia in critically ill patients. *Clin Oral Investig* **25**, 1217 (2021).

8. Cook, A., Norwood, S. & Berne, J. Ventilator-associated pneumonia is more common and of less consequence in trauma patients compared with other critically ill patients. *J Trauma* **69**, 1083–1091 (2010).

9. Shi, Z. *et al.* Oral hygiene care for critically ill patients to prevent ventilator-associated pneumonia. *Cochrane Database Syst Rev* **2013**, (2013).

10. Klompas, M. Does This Patient Have Ventilator-Associated Pneumonia? *JAMA* **297**, 1583–1593 (2007).

11. Fernando, S. M. *et al.* Diagnosis of ventilator-associated pneumonia in critically ill adult patients-a systematic review and meta-analysis. *Intensive Care Med* **46**, 1170–1179 (2020).
